# Chemopreventive effect of cactus *Opuntia ficus indica *on oxidative stress and genotoxicity of aflatoxin B1

**DOI:** 10.1186/1743-7075-8-73

**Published:** 2011-10-18

**Authors:** Dalel Brahmi, Chayma Bouaziz, Yousra Ayed, Hédi Ben Mansour, Lazhar Zourgui, Hassen Bacha

**Affiliations:** 1 Laboratory of Research on Biologically Compatible Compounds, Faculty of Dentistry, Rue Avicenne, 5019 Monastir, Tunisia; 2 Research unit of Macromolecular Biochemistry & Genetic, Faculty of Sciences Gafsa - 2112 Gafsa, Tunisia; 3 Laboratoire de Biochimie, Faculté de Pharmacie, Monastir, Rue Avicenne 5000 Monastir, Tunisia; 4 Higher Institute of Applied Biology ISBAM Medenine university of Gabes, Tunisia

**Keywords:** Cactus, Aflatoxin B1, Oxidative Stress, Genotoxicity, Hepatopretective

## Abstract

**Background:**

Aflatoxin B1 (AFB1) is potent hepatotoxic and hepatocarcinogenic agent. In aflatoxicosis, oxidative stress is a common mechanism contributing to initiation and progression of hepatic damage. The aim of this work was to evaluate the hepatoprotective effect of cactus cladode extract (CCE) on aflatoxin B1-induced liver damage in mice by measuring malondialdehyde (MDA) level, the protein carbonyls generation and the heat shock proteins Hsp 70 and Hsp 27 expressions in liver. We also looked for an eventual protective effect against AFB1-induced genotoxicity as determined by chromosome aberrations test, SOS Chromotest and DNA fragmentation assay. We further evaluated the modulation of p53, bax and bcl2 protein expressions in liver.

**Methods:**

Adult, healthy balbC (20-25 g) male mice were pre-treated by intraperitonial administration of CCE (50 mg/Kg.b.w) for 2 weeks. Control animals were treated 3 days a week for 4 weeks by intraperitonial administration of 250 μg/Kg.b.w AFB1. Animals treated by AFB1 and CCE were divided into two groups: the first group was administrated CCE 2 hours before each treatment with AFB1 3 days a week for 4 weeks. The second group was administrated without pre-treatment with CCE but this extract was administrated 24 hours after each treatment with AFB1 3 days a week for 4 weeks.

**Results:**

Our results clearly showed that AFB1 induced significant alterations in oxidative stress markers. In addition, it has a genotoxic potential and it increased the expression of pro apoptotic proteins p53 and bax and decreased the expression of bcl2. The treatment of CCE before or after treatment with AFB1, showed (i) a total reduction of AFB1 induced oxidative damage markers, (ii) an anti-genotoxic effect resulting in an efficient prevention of chromosomal aberrations and DNA fragmentation compared to the group treated with AFB1 alone (iii) restriction of the effect of AFB1 by differential modulation of the expression of p53 which decreased as well as its associated genes such as bax and bcl2.

**Conclusion:**

We concluded that CCE might have a hepatoprotective effect against aflatoxicosis in mice, probably acting by promoting the antioxidant defence systems.

## Background

Primary liver cancer, also known as hepatocellular carcinoma (HCC), happens to be the sixth most common cancer as well as the third leading cause of cancer mortality in the world [1]. The incidence of HCC is on the rise in multiple geographic areas, including Asia Pacific, sub-Saharan Africa, Southern Europe as well as North America. It has been estimated that there will be more than 22,000 new cases and about 18,000 deaths in the United States in 2009 due to liver cancer which represents about 4% of cancer mortality in this country [2]. The vast majority of HCC cases are attributable to underlying infections caused by the hepatitis B and C viruses [3], nevertheless several other risk factors, namely alcoholism, as well as dietary carcinogens, such as aflatoxins and nitrosamines are also involved in its etiology [4,5].

In this work we are interested on aflatoxins (AF), a group of mycotoxins which are common contaminants in a wide variety of food. AF are produced as secondary metabolites by Aspergillus flavus and Aspergillus parasiticus fungi. AF not only contaminate our food stuffs but are also found in edible tissues, milk and eggs after consumption of contaminated feed by farm animals [6,7]. AF are the collective term for four major naturally occurring secondary compounds (B1, B2, G1 and G2). Aflatoxins B1 (AFB1) is the most potent of these toxins, which has hepatotoxic and hepatocarcinogenic properties [8]. The International Agency for Research on Cancer IARC classified AFB1 and mixtures of aflatoxins as Group 1 carcinogens [9]. The liver is the main target organ for AF and chronic exposure to low levels in foodstuffs causes liver fibrosis and primary liver cancer [10]. It is metabolized in the liver producing the formation of highly reactive chemical intermediaries. The carcinogenic mechanism of AFB1 has been extensively studied. It has been shown that AFB1 is activated by hepatic cytochrome P450 enzyme system to produce a highly reactive intermediate, AFB1-8,9-epoxide, which subsequently binds to nucleophilic sites in DNA, and the major adduct 8,9-dihydro-8 (N7guanyl)- 9-hydroxy-AFB1 (AFB1 N7-Gua) is formed [11,12]. In addition its genotoxic proprieties, it can induce oxidative stress both "*in vivo*" and "*in vitro*" [13,14]. In view of the limited treatment and grave prognosis of liver cancer, preventive control approaches, notably chemoprevention, have been considered as one of the best strategies in lowering the current morbidity and mortality associated with HCC [15,16]. A detailed understanding of the pathogenesis of HCC holds the promise of finding an effective and novel strategy for the chemoprevention and treatment of liver cancer. Recently, natural foods and food derived antioxidants such as vitamins and phenolic phytochemicals have received growing attention, because they are known to function as chemopreventive agents against oxidative damage and genotoxicity. Fruits, vegetables and herb medicines contain many antioxidant compounds, including carotenoids, thiols vitamins such as ascorbic acid, tocopherols, flavonoids, and other phenolics [17]. Active principles with diverse chemical structures have been isolated from plants reportedly possessing hepatoprotective effects. Cactus *Opuntia ficus indica*, a member of the Cactaceae family, is an important forage crop for livestock in many arid and semi-arid regions of the world. It is widely distributed in Mexico and in all American hemispheres as well as in Africa and in the Mediterranean basin [18]. Fruit and cladode of this plant yield high values of important nutrients such as minerals, vitamins as well as further antioxidants [19-22]. Besides, several studies have reported its efficiency in the treatment of several diseases. These fruits have shown several effect such as antiulcerogenic [23], antioxidant [23-25], anticancer [26] and hepatoprotective activities [27]. Different parts of *Opuntia ficus-indica *are used in the traditional medicine of several countries: the cladodes are utilized for treatment of ulcers, rheumatic pain, wounds, fatigue; in addition, in our laboratory a recent study showed the potential antigenotoxic activities of cactus cladodes against single dose of the mycotoxin zearalenone (ZEN), a potent estrogenic metabolite [28]. These data have made cactus pear fruits and cladodes perfect candidates for cytoprotective investigations.

The aim of the present study was to find out the eventual protective effect of CCE against AFB1-induced hepatotoxicity *in vivo *using Balb/c mice. We evaluated the oxidative status, the mutagenic and the genotoxic potential of AFB1 alone or jointly with CCE. To this end, we measured MDA concentrations, the protein carbonyls generation and heat shock protein (Hsp70 and Hsp 27) expressions. We also evaluated chromosome aberrations, DNA fragmentation, mutagenic activity, p53, bax and bcl2 protein expressions.

## Materials and methods

### Chemicals

AFB1 was obtained from Sigma Chemical Co. (USA). Dimethyl sulfoxide (DMSO) was obtained from Sigma Chemical Co. (St Louis, MO, USA). Nitro blue tetrazolium (NBT) and 5-bromo-4-chloro-3-indolyl phosphate disodium salt (BCIP) were from Sigma Aldrich, France. Goat anti-mouse alkaline phosphatase conjugate antibody, mouse anti-Hsp 70 and anti-Hsp 27 monoclonals antibody (SPA-80) were from Stressgen, USA. Mouse monoclonal anti-p53, anti-bax and anti-bcl2 and the secondary antibody (phosphatase-conjugated) were from Invitrogen. Gen Elute "Mammalian genomic DNA Miniprep Kit sufficient for 70 purifications" was purchased from Sigma AIdrich, USA. All other chemicals used were of the highest grade available from commercial sources.

### Extract of cactus cladodes

Young cactus cladodes of *Opuntia ficus-indica *(2-3 weeks of age) collected from the local area were washed with water chopped into small pieces and then pressed using a hand-press, homogenized in 10 mM Tris-HCl, pH 7.4 at 4°C and centrifuged 30 min at 3500 g at 4°C. The supernatant was collected, dried and stored at -20°C.

### Animals and treatments

Adult, healthy balbC (20-25 g) male mice provided from an animal breeding centre (SEXAL, St. Doulchard, France following the agreement of the Ethics Committee named National committee of Medical ethics CNEM, BP 74 - Pasteur Institute Tunis 1002 TUNISIA) were used. The animals were kept for acclimatization 1 week under constant conditions of temperature and a light/dark cycle of 12 h: 12 h. Animals had free access to standard granulated chow and drinking water. Animals were pre-treated by intraperitonial administration of CCE (50 mg/Kg.b.w) for 2 weeks. Control animals were treated 3 days a week for 4 weeks by intraperitonial administration of 250 μg/Kg.b.w AFB1. Animals treated by AFB1 and CCE were divided into two groups: the first group was administrated CCE 2 hours before each treatment with AFB1 3 days a week for 4 weeks. The second group was administrated without pre-treatment with CCE but the extract was administrated 24 hours after each treatment with AFB1 3 days a week for 4 weeks.

All animals were divided in 9 groups of 6 animals per group and treated as follows:

Group 1: Mice given H2O (100 μl)

Group 2: Mice given DMSO/H2O (1:1, v: v)

Group 3: Mice given CCE 50 mg/Kg b.w

Group 4: Mice given AFB1 250 μg/Kg b.w for15 days treatment

Group 5: Mice given AFB1 250 μg/Kg b.w + CCE 50 mg/Kg b.w (before 15 days treatment by AFB1)

Group 6: Mice given AFB1 250 μg/Kg b.w + CCE 50 mg/Kg b.w (after 15 days treatment by AFB1)

Group 7: Mice given AFB1 250 μg/Kg b.w for 30 days treatment

Group 8: Mice given AFB1 250 μg/Kg b.w + CCE 50 mg/Kg b.w (before 30 days treatment by AFB1)

Group 9: Mice given AFB1 250 μg/Kg b.w + CCE 50 mg/Kg b.w (after 30 days treatment by AFB1).

### Preparation of liver extracts

Livers of mice were homogenized with a Potter (glass-Teflon) in the presence of 10 mM Tris-HCl, pH 7.4 at 4°C and centrifuged at 4000 rpm for 30 min at 4°C. The supernatant was collected for analysis and the protein concentration was determined in liver extract using Protein BioRad assay [29].

### Evaluation of lipid peroxidation status

Lipid peroxidation was determined indirectly by measuring the production of MDA in the liver extracts following the method of Aust et al. (1985) [30]. Briefly, 200 μl of liver extracts were mixed with 150 μl of TBS (Tris 50 mM and NaCl 150 mM, pH 7.4) and 250 μl TCA-BHT (20% TCA and BHT 1%). The mixture was vigorously vortexed and centrifuged at 1500 g for 10 min. 400 μl of the supernatant were added with HCl 0.6 N and 320 μl Tris-TBA (Tris 26 mM and TBA 120 mM), the content was mixed and incubated 10 min at 80°C. The absorbance was measured at 535 nm. The optic density corresponding to the complex formed with the TBA-MDA is proportional to the concentration of MDA and to the lipid peroxide. The concentration of μmol of MDA/mg of proteins is calculated from the absorbance at 530 nm using the molar extinction coefficient of MDA 1.56 × 10^5 ^M^-1 ^cm^-1^.

### Protein carbonyl assay

Protein carbonyls content was determined as described by Mercier et al. (2004) [31] in liver homogenates by measuring the reactivity of carbonyl groups with 2,4-dinitrophenylhydrazine (2,4-DNPH). Thus, 200 μl of supernatant of liver were placed in glass tubes. 800 μl of 10 mM DNPH in 2.5 M HCl were added. Tubes were left for 1 h of incubation at room temperature in the dark. Samples were vortexed every 15 min. Then 1 ml of 20% TCA was added to samples, and the tubes were left in ice bucket for 10 min and centrifuged for 5 min at 4000 rpm to collect the protein precipates and the supernatants were discarded. Next, another wash is performed using 1 ml of 10% TCA, and protein pellets are broken mechanically with the aid of glass rod. Finally, the pellets are washed with 1 ml of ethanol-ethyl acetate (1:1, v/v) to remove the free DNPH. The final precipitates are dissolved in 500 μl of guanidine hydrochloride 6 M and are left for 10 min at 37°C with general vortex mixing. Any insoluble materials are removed by additional centrifugation. Protein carbonyls concentration was determined from the absorbance at 370 nm, applying the molar extinction coefficient of 22.0 Mm ^-1 ^cm^-1^. A range of nmoles of carbonyls per ml is usually obtained for most proteins and is related to the protein content in the pellets.

### Protein extraction and Western blot analysis

Equal amounts of proteins (20 μg) were separated by 12% SDS-polyacrylamide gel electrophoresis. Separated proteins were electro-blotted on nitrocellulose membrane in the transfer buffer (10 ml Tris-base, pH 8.3, 96 mM glycine and 10% methanol). The membrane was then blocked in TBS (20 mM Tris-HCl, Ph 7.5, 500 mM sodium chloride) containing 5% of BSA, washed in TTBS (TBS containing 0.3% Tween 20) and probed with an antibody for anti-Hsp 70, anti-Hsp 27, anti-p53, anti-bax and anti-bcl2 at a 1:1000 dilution for 6 h at room temperature. The membrane was washed and incubated with goat anti-mouse alkaline phosphate conjugated at a 1:3000 dilution for 1 h. finally, the membrane was washed and the chromogenic substrate BCIP/NBT was added to localize antibody binding. Hsp 70, Hsp 27, p53, bax and bcl2 levels were then determined by computer-assisted densitometric analysis (Densitometer, GS-800, BioRad Quantity One).

### Chromosome aberrations assay

24 hours before sacrifice, animals were given a suspension of yeast powder (100 mg/500 μl) to accelerate mitosis of bone-marrow cells. Vinblastine (200 μl; 250 μg/ml) was injected into the animals 45 min before sacrifice in order to block dividing cells in metaphasis. Bone marrow cells from femurs and tibias were collected, subjected to hypotonic shock (KCl 0.075 M) and fixed three times using methanol-acetic acid [32]. The cells were spread on glass slides that were blazed on a flame for 5 s, then air-dried for conservation at room temperature and finally stained by 4% dilution of Giemsa reagent in water for 15 min. After coding of the slides, the chromosomes of 100 cells in metaphase were examined for abnormalities at a magnification of 1000 × using an optical microscope (Carl Zeiss, Germany).

This was done for each one of three replicates (300 metaphases per dose level) for negative controls, positive controls and treated groups. Chromosome aberrations were identified according to criteria described by Savage (1975) [33]. Metaphases with chromosome breaks, gaps, rings and centric fusions (robertsonian translocation) were recorded and expressed as percentage of total metaphases per group.

### Detection of fragmented DNA by agarose gel electrophoresis

Mammalian tissues (livers) were lysed with a chaotropic salt-containing buffer to ensure denaturation of macromolecules. DNA is bound to the spin column membrane and the remaining lysate is removed by centrifugation. A filtration column is used to remove cell debris, after washing to remove contaminants; the DNA is eluted with buffer into a collection tube. The pellet was rinsed with 70% ethanol, dried at room temperature for 2 h and resuspended in 200 μl of TE (20 mM Tris-HCl pH 8.0,1 mM EDTA). Loading buffer was added to 10 μg of DNA for each treatment, and the samples were analyzed by electrophoresis on a 1% agarose gel (1 h at 80 V/30 mA) with a TBE running buffer (44 mM Tris-HCl,44 mM boric acid, 50 mM EDTA, pH8.0).

Quantitative analysis of DNA samples was performed by UV spectrophotometry (1 OD = 50 μg DNA ml-1, max = 258 to 260 nm). Each DNA sample was prepared and stored at -80°C prior to use.

### Activation mixture

The S9 microsome fraction was prepared from the liver of rats treated with Aroclor 1254 [34]. The composition of the activation mixture is the following per 10 ml of S9 mix: salt solution (1.65 M KCl + 0.4 M MgCl2 - 6H2O) 0.2 ml; G6P (1 M) 0.05 ml; NADP (0.1 M) 0.15 ml; Tris buffer (0.4 M pH7.4) 2.5 ml; Luria broth medium 6.1 ml; S9 fraction 1 ml.

### SOS chromotest

The SOS chromotest assay is a bacterial test for detecting DNA damaging agent. It was employed to determine the effect of cactus cladode extract on the genotoxicity of aflatoxin B1 (direct acting mutagen) induced genotoxicity. The SOS chromotest with Escherichia coli PQ37strain was performed according to the procedure described by Quillardet and Hofnung (1985) [35]. The genotype of this strain is: F-thr leu his-4 pyrD thi galE galK lacDU169 Srl300Tn10 rpoB rpsL uvrA rfa trp Muc + sfiA::Mud (Ap, lac) cts. An exponential-phase culture of E. coli PQ37 was grown at 37°C in LB medium to an approximate cell density of 2.10^8 ^cell/ml supplemented with ampicillin (20 μg/ml). One ml of this culture was diluted with 9 ml of fresh medium; Positive controls were prepared by exposure of the bacteria to AFB1. After 2 h of incubation at 37°C, with shaking, 300 μl samples were used for assaying β- galactosidase (β-gal) and alkaline phosphatase (AP) activities. In this assay, the β-galactosidase synthesis (lacZ gene) is dependent on sfiA activation and is used to measure induction of SOS repair system. The activity of the constitutive enzyme alkaline phosphatise was used as a measure of protein synthesis and toxicity.

Enzyme activities were assessed spectrophotometrically. The SOS induction factor (IF) in treated cells was obtained by comparing β-galactosidase and alkaline phosphatase activities in treated and untreated cells. The result was considered positive when the IF for β-galactosidase activity was > 2.0. For evaluation of the protective effect of CCE on the induction of the SOS response by AFB1 (in the presence of the S9 activation mixture), 10 μl of AFB1 (10 μg/assay) were added into tubes with 10 μl of the tested concentration of CCE.

Antigenotoxicity was expressed as percentage inhibition of genotoxicity induced by AFB1 according to the formula: % = 100 - (IF1 - IF0/IF2 - IF0) *100

Where IF1 is the induction factor in the presence of the test compound and the genotoxin, IF2 the induction factor in the absence of the test compound and in the presence of the genotoxin, and IF0 is the induction factor of the negative control. Data were collected as a mean ± S.D. of experiments.

### Statistical analysis

Each experiment was carried out in triplicates. Data are expressed as means ± standard deviation (S.D.). Differences between groups were determined using one-way ANOVA with Bonferroni's post multiple comparisons, Expression of Hsp 70, Hsp 27, p53, bax and bcl2 were determined by Kruskal-Wallis Test. The level of significance was accepted with P < 0.05 was used for statistical analysis.

## Results

### Effect of CCE on oxidative stress induced by AFB1

#### Evaluation of lipid peroxidation status

**Figure 1:**
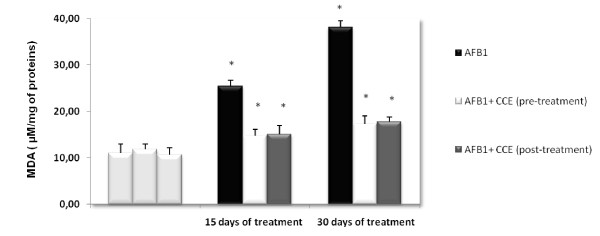
**Lipid peroxydation as determined by MDA level in liver of Balb/c mice exposed to AFB1 (250 μg/Kg b.w.) for 15 days then 30 days and prevention by cactus cladode extract (50 mg/Kg b.w) before or after AFB1 administration**. Results were expressed as means ± S.D. from independent experiments. (*) indicated significant difference (p < 0.05) from control.

Results of the effect of AFB1 alone and jointly with CCE on the induction of lipid peroxidation in liver as determined by MDA level are shown in Figure 1, AFB1 induced a significant increase in MDA formation as compared to control groups especially on day 30. The MDA level increased from a basal level of 11.05 ± 0.25 lM/mg of protein to reach 25.50 ± 0.15 l M/mg of protein and 38.15 ± 0.75 l M/mg on days 15 and 30 respectively. The increase in MDA levels was about two folds as compared to the control group (p < 0.05). Interestingly, when animals were treated with CCE (50 mg/kg b.w) a sharp decrease in MDA level was noticed in both 15 day and 30 day times. For a pre and post-treatment effect, MDA level has decreased significantly to reach the control level.

#### Protein carbonyl assay

**Figure 2:**
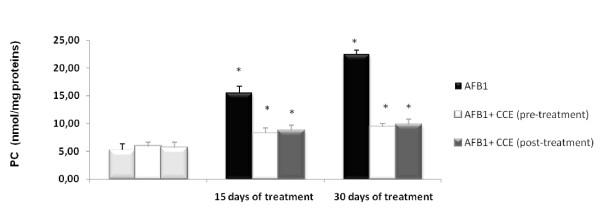
**Concentrations of protein carbonyls in liver of treated mice with AFB1 (250 μg/Kg b.w.)**. Cytoprotective effects of cactus cladodes extract (50 mg/Kg b.w.) before or after treatment with AFB1. Data are exposed as the means ± S.D.

Protein carbonyls formation, indicative of severe protein oxidation was assayed in liver homogenates and results are illustrated in Figure 2. AFB1 generates protein carbonyls formation as compared to control groups in liver extracts. Indeed, the protein carbonyls level increases from basal value of 5.25 ± 0.10 nmol/mg of protein in control group to reach 15.50 ± 0.04 nmol/mg of protein and 22.45 ± 0.03 nmol/mg of protein in AFB1 treated group after respectively 15 and 30 days of treatment. The cactus cladodes extract remarkably decreased protein carbonyls formation induced by AFB1 (250 μg/Kg b.w.) by 60% in liver extracts.

#### Determination of Hsp70 and Hsp27expressions

**Figure 3:**
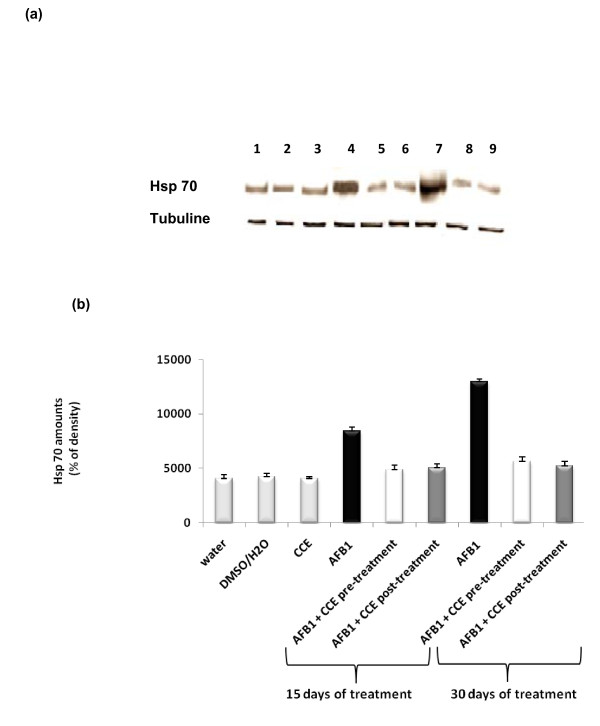
**Immunoblot (a) and densitometric (b) analysis of Hsp 70 in liver of control and treated animals**. The protein was separated on 12% SDS-PAGE and blotted with anti-Hsp70 antibody. The intensity of the protein band was scanned by densitometry. Results are significantly different as compared to controls (p < 0.005). The results are representative of nine independent experiments: (1) Animals treated by 100 μl H2O. (2) Animals treated by mixture of DMSO/H2O (1:1; v:v). (3) Animals treated by CCE 50 mg/Kg b.w. (4) Animals treated 15 days by AFB1 250 μg/Kg b.w. (5) Animals treated by CCE 50 mg/Kg b.w before AFB1 250 μg/Kg b.w exposure for 15 days treatment. (6) Animals treated by CCE 50 mg/Kg b.w after AFB1 250 μg/Kg b.w exposure for 15 days treatment. (7) Animals treated 30 days by AFB1 250 μg/Kg b.w. (8) Animals treated by CCE 50 mg/Kg b.w before AFB1 250 μg/Kg b.w exposure for 30 days treatment. (9) Animals treated by CCE 50 mg/Kg b.w after AFB1 250 μg/Kg b.w exposure for 30 days treatment.

**Figure 4:**
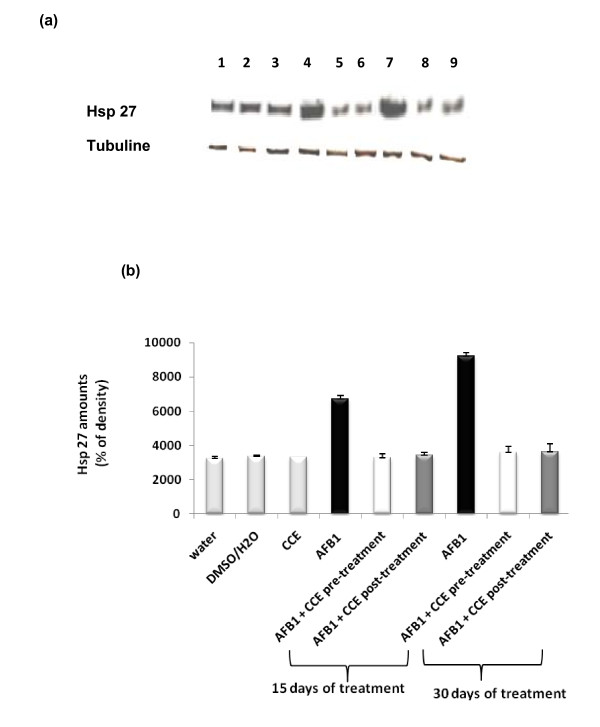
**Immunoblot (a) and densitometric (b) analysis of Hsp 27 in liver of control and treated animals**. The protein was separated on 12% SDS-PAGE and blotted with anti-Hsp 27 antibody. The intensity of the protein band was scanned by densitometry. Results are significantly different as compared to controls (p < 0.005).

Figures 3a and 3b show the western blotting and densitometry analysis of hsp70 expression in livers of control and treated animals. AFB1-exposed mice showed significantly increased expression of hsp70 after 15 days and remarkably after 30 days exposure on liver extract compared to control groups. Administration of CCE before or after AFB1 exposure decreased significantly the hsp70 expression. This decrease reached the basal expression observed in control groups. Similar results were found for Hsp 27 expression (Figures 4a and 4b)

### Effect of CCE on DNA damage induced by AFB1

#### Eventual prevention of *AFB1-induced *chromosome aberrations by CCE

**Figure 5:**
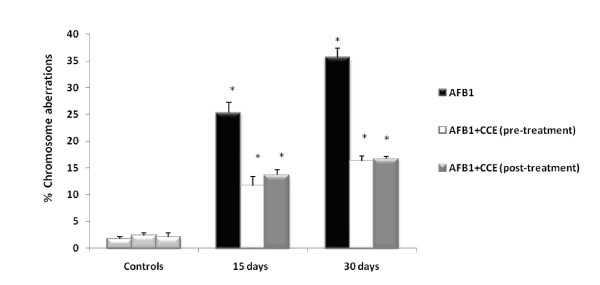
**Effect of cactus cladodes on chromosomal aberrations in bone marrow cells of AFB1 treated Balb/c mice**. Results are expressed as mean ± standard deviation (n = 3). (*) indicated significant difference (p < 0.05) from control.

Genotoxicity of AFB1 was assessed through test of chromosome aberrations in mice bone marrow cells. Results of the visual scoring of total DNA damage induced by AFB1 are shown in Figure 5. We observed that animals treated with AFB1 alone (250 μg/kg b.w) showed a significant increase in chromosome aberrations in bone marrow cells especially on day 30 with 35% of chromosome aberrations. Control groups which were treated with H2O, H2O/DMSO or CCE showed a similar basal and low percentage of total chromosome aberrations (respectively 1.67 ± 0.18; 2.33 ± 1.56 and 2 ± 0.15). But we remarked that the coadministration of cactus before or after AFB1 treatment decreased significantly the total chromosomal aberrations. Meanwhile, the protection by cactus extract was not total; it reached 60% (Figure 5).

#### Eventual prevention of *AFB1-induced *DNA fragmentation by CCE

**Figure 6:**
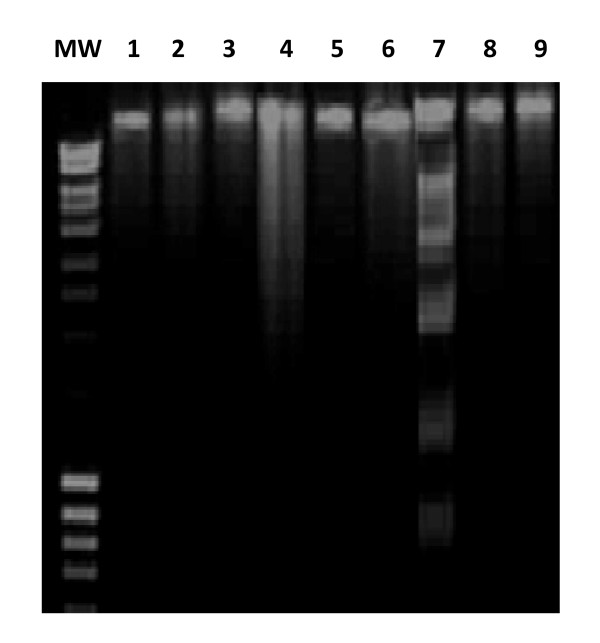
**DNA fragmentation of mice liver extracts induced by AFB1 and prevention of CCE revealed by agarose gel electrophoresis**. The results are representative of nine lines: MW: Markers (MW = 1 Kb). (1) Animals treated by 100 μl H2O. (2) Animals treated by mixture of DMSO/H2O (1:1; v:v). (3) Animals treated by CCE 50 mg/Kg b.w. (4) Animals treated 15 days by AFB1 250 μg/Kg b.w. (5) Animals treated by CCE 50 mg/Kg b.w before AFB1 250 μg/Kg b.w exposure for 15 days treatment. (6) Animals treated by CCE 50 mg/Kg b.w after AFB1 250 μg/Kg b.w exposure for 15 days treatment. (7) Animals treated 30 days by AFB1 250 μg/Kg b.w. (8) Animals treated by CCE 50 mg/Kg b.w before AFB1 250 μg/Kg b.w exposure for 30 days treatment. (9) Animals treated by CCE 50 mg/Kg b.w after AFB1 250 μg/Kg b.w exposure for 30 days treatment.

Results obtained after agarose gel electrophoresis are shown in Figure 6; No specific DNA fragments were observed for control groups (lanes 1, 2, 3). Animals treated by AFB1 (250 μg/kg b.w) for 15 days and 30 days (lanes 4 and 7 respectively) showed a significant DNA fragmentation in liver cells. Simultaneous treatment of mice with CCE before or after AFB1 exposure for 15 days and 30 days showed a significant restoration of DNA (lanes 5, 6, 8 and 9 respectively).

#### The SOS Chromotest assay

Experiments realized with CCE revealed no genotoxicity induction in so far as the induction factor is not higher than 1.5. While experiment with AFB1 gave the maximum of genotoxicity with IF = 4.24. The inhibitory effect of the tested product on the genotoxicity induced by AFB1using the SOS chromotest is illustrated by table 1. This study shows that CCE present an antigenotoxic effect at the tested concentrations. Indeed CCE significantly decreases the IF of AFB1 by 64%.

**Table 1:** Genotoxic activity of CCE and AFB1 by the SOS Chromotest in the presence of E.coli PQ37.

Extract	β-gal (U)	AP (U)	IF
**NC**	1,65	1,9	
**AFB1**	9,21	2,5	4,24
**CCE**	1,1	1,7	0,73
**AFB1+CCE**	1,56	1,25	1,42

β-gal: β-galactosidase; AP: alkaline phosphatase; U: enzyme units; IF: induction factor; NC: negative control (non treated cells).

### Effect of CCE on apoptose status

#### Determination of p53 expression

**Figure 7:**
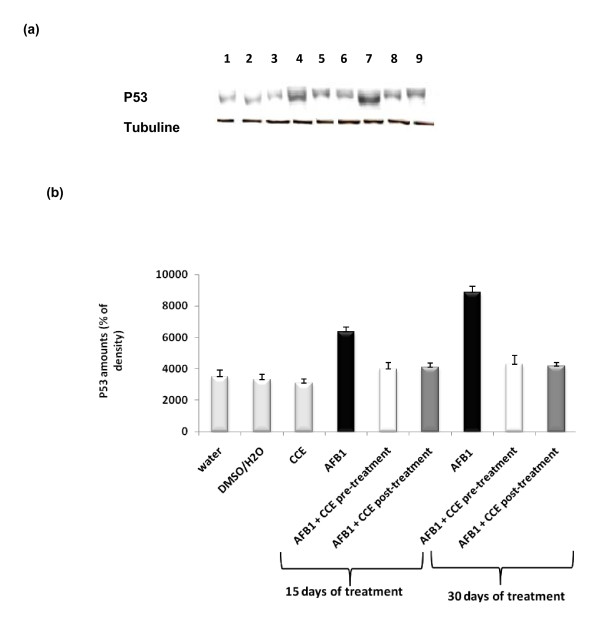
**Immunoblot (a) and densitometric (b) analysis of p53 in liver of control and treated animals**. The protein was separated on 12% SDS-PAGE and blotted with anti-p53 antibody. The intensity of the protein band was scanned by densitometry. Results are significantly different as compared to controls (p < 0.005). The results are representative of nine independent experiments: (1) Animals treated by 100 μl H2O. (2) Animals treated by mixture of DMSO/H2O (1:1; v:v). (3) Animals treated by CCE 50 mg/Kg b.w. (4) Animals treated 15 days by AFB1 250 μg/Kg b.w. (5) Animals treated by CCE 50 mg/Kg b.w before AFB1 250 μg/Kg b.w exposure for 15 days treatment. (6) Animals treated by CCE 50 mg/Kg b.w after AFB1 250 μg/Kg b.w exposure for 15 days treatment. (7) Animals treated 30 days by AFB1 250 μg/Kg b.w. (8) Animals treated by CCE 50 mg/Kg b.w before AFB1 250 μg/Kg b.w exposure for 30 days treatment. (9) Animals treated by CCE 50 mg/Kg b.w after AFB1 250 μg/Kg b.w exposure for 30 days treatment.

Figures 7a and 7b show the Western blotting and densitometry analysis of p53 expression in liver of controls and treated animals. After 15 days and 30 days exposure to AFB1 alone, p53 expression was found to be significantly increased compared to controls but it decreased by CCE pre or post-treatment. The CCE treated group did not have any significant effect on the expression of p53.

#### Determination of bax expression

**Figure 8:**
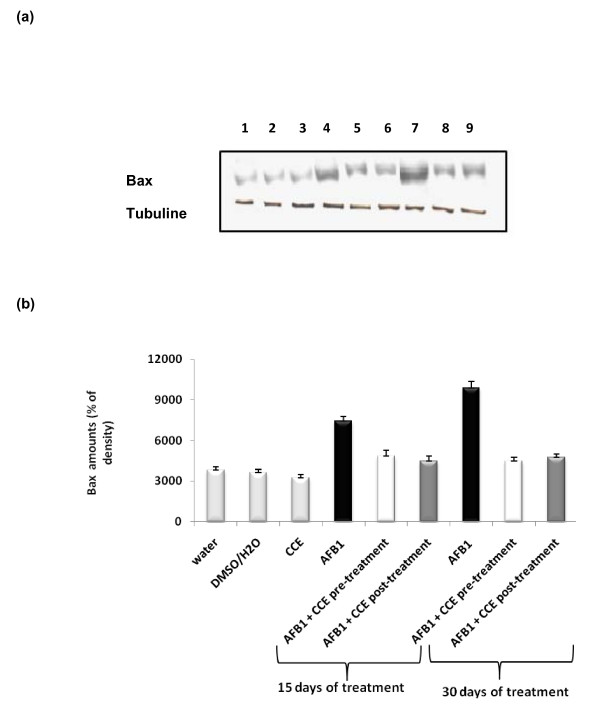
**Immunoblot (a) and densitometric (b) analysis of bax in liver of control and treated animals**. The protein was separated on 12% SDS-PAGE and blotted with anti-bax antibody. The intensity of the protein band was scanned by densitometry. Results are significantly different as compared to controls (p < 0.005). The results are representative of nine independent experiments: (1) Animals treated by 100 μl H2O. (2) Animals treated by mixture of DMSO/H2O (1:1; v:v). (3) Animals treated by CCE 50 mg/Kg b.w. (4) Animals treated 15 days by AFB1 250 μg/Kg b.w. (5) Animals treated by CCE 50 mg/Kg b.w before AFB1 250 μg/Kg b.w exposure for 15 days treatment. (6) Animals treated by CCE 50 mg/Kg b.w after AFB1 250 μg/Kg b.w exposure for 15 days treatment. (7) Animals treated 30 days by AFB1 250 μg/Kg b.w. (8) Animals treated by CCE 50 mg/Kg b.w before AFB1 250 μg/Kg b.w exposure for 30 days treatment. (9) Animals treated by CCE 50 mg/Kg b.w after AFB1 250 μg/Kg b.w exposure for 30 days treatment.

AFB1 induces the expression of bax in liver as evidenced by immunoblotting illustrated in Figure 8a, which was further, confirmed by results of scanning densitometry (Figure 8b). The administration of CCE before and after AFB1 exposure for 15 and 30 days treatment decreased the amounts of bax (Figure 8a and 8b). The CCE treated group did not show any significant modification on the expression of bax.

#### Determination of bcl2 expression

**Figure 9:**
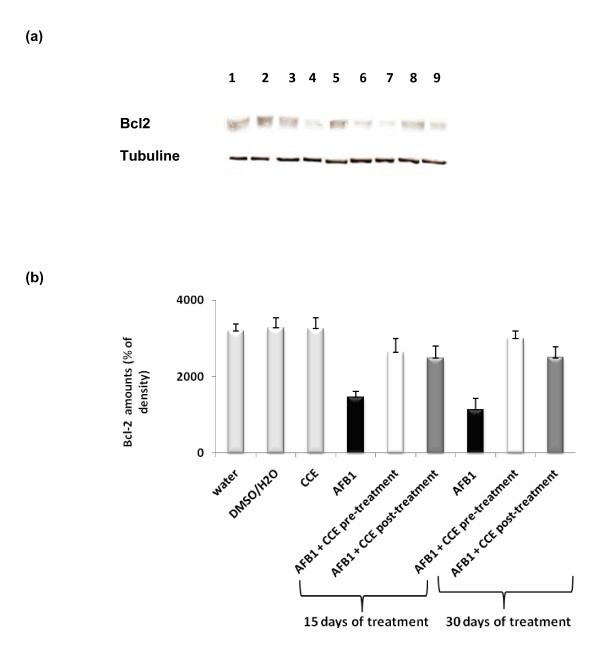
**Immunoblot (a) and densitometric (b) analysis of bcl2 in liver of control and treated animals**. The protein was separated on 12% SDS-PAGE and blotted with anti-bcl2 antibody. The intensity of the protein band was scanned by densitometry. Results are significantly different as compared to controls (p < 0.005). The results are representative of nine independent experiments: (1) Animals treated by 100 μl H2O. (2) Animals treated by mixture of DMSO/H2O (1:1; v:v). (3) Animals treated by CCE 50 mg/Kg b.w. (4) Animals treated 15 days by AFB1 250 μg/Kg b.w. (5) Animals treated by CCE 50 mg/Kg b.w before AFB1 250 μg/Kg b.w exposure for 15 days treatment. (6) Animals treated by CCE 50 mg/Kg b.w after AFB1 250 μg/Kg b.w exposure for 15 days treatment. (7) Animals treated 30 days by AFB1 250 μg/Kg b.w. (8) Animals treated by CCE 50 mg/Kg b.w before AFB1 250 μg/Kg b.w exposure for 30 days treatment. (9) Animals treated by CCE 50 mg/Kg b.w after AFB1 250 μg/Kg b.w exposure for 30 days treatment.

Figure 9a and 9b shows the western blotting and densitometry analysis of bcl2 expression in liver of controls and treated animals. After 15 days and 30 days exposure to AFB1 alone, anti-apoptotic protein bcl2 expression was found to be significantly decreased by 25% and 35% respectively after 15 and 30 days of AFB1 treatment compared to controls, but it increased before and after treatment by CCE. The CCE treated group did not show any significant modification on the expression of bcl2.

## Discussion

Increasing attention has been given to the study of natural products, which may counteract the detrimental effects of environmental toxic compounds and prevent multiple human diseases. In this line, different types of fruits and vegetables have been re-evaluated and recognized as valuable sources of nutraceuticals. According to several studies, cactus pear (Opuntia ssp.) yield high values of important nutrients and exhibit antioxidant functions [25,22]. In this work we evaluated the effect of CCE 50 mg/kg b.w tested in Balb/c by monitoring its effects on oxidative stress, genotoxicity and cell death pathway induced by sub-chronic treatment by AFB. We have chosen this dose based on previous reports which have proved its efficiency on preventing toxicity induced by the mycotoxin zearalenone [28]. Exposure to low levels of aflatoxins is one of the major risk factors in the etiology of human hepatocellular carcinoma. AFB1 is a potent hepatocarcinogen when given sub-chronically at a low level. Hence, we have chosen treatment by 250 μg/Kg b.w of AFB1 in sub-chronic condition [36-38]. To evaluate the oxidative status, we looked for an eventual lipid peroxidation. Determination of malondialdehyde (MDA) is considered to be an excellent index of lipid oxidation. The MDA is the end product of lipoperoxydation, considered as a late biomarker of oxidative stress and cellular damage [39,40]. In the present study, exposure to AFB1 (250 μg/kg b.w) induced a marked increase in MDA level in liver (Figure 1). The oxidative damage caused by aflatoxin is considered to be the main mechanism leading to the subsequent hepatotoxicity [41]. AFB1 may disturb the integrity of cell membranes through stimulating phospholipid A2 to initiate lipid peroxidation in cells [42]. Results of others researches supported the earlier finding that AFs-induced oxidative stress and increased lipid peroxidation [43]. The pre and post-administration of CCE with AFB1 significantly reduced this oxidative effect which dropped to the control level.

To further assess AFB1 oxidative induced damages in Balb/c mice, the protein carbonyls generation was monitored. Protein carbonylation is a sign of irreversible oxidative damage, often leading to a loss of protein function, which may have lasting detrimental effects on cells and tissues [44,45]. Our results clearly showed that AFB1 induced a marked increase in protein carbonyls generation in liver extracts which was significantly reduced with cactus extract in liver (Figure 2). Finally, to further study of oxidative stress in AFB1 induced toxicity, we choose to monitor early markers of oxidative stress. Nonspecific cellular oxidative damage is often observed during toxicity [46]. In fact, based on the analysis of MDA, protein carbonyls only (presumed late biomarkers of oxidative damage), it is difficult to determine whether severe oxidative stress is the cause or the consequence of cellular toxicity. Thus, levels of early markers of oxidative stress including antioxidant enzymes and Hsp, may be altered in the presence of lower levels of oxidative stress and before the biomarkers of severe oxidative stress attributed to cytotoxicity appear.

Hsps are induced and play a key role in cell protection and repair [47,48]. This protein expression is triggered by structural damage caused to cell proteins mainly thiol oxidation and general perturbations of the cellular redox status level [49-51]. Several published data have reported that many sources of oxidative stress can lead to the up-regulation of the Hsp 70 as well as small Hsps such as Hsp 27 at levels where overt oxidative damage is not observed [52,53]. Our results clearly demonstrated that treatment by AFB1 alone induced a sharp elevation in the expression level of both Hsp70 and Hsp 27 in liver of mice after 15 days and especially after 30 days treatment. Interestingly, when animals were treated by CCE before or after administration of AFB1, a sharp decrease of Hsp 70 and Hsp 27 levels was observed (Figure 3a, b and 4a, b). These results are in agreement with findings of Zourgui et al. (2008) [54] reporting that CCE was effective in the protection against acute toxicity induced by mycotoxin ZEN which increased Hsp 70 and Hsp 27 expressions in liver and kidney extracts. CCE ability to prevent and protect against oxidative damage is certainly associated to the presence of several antioxidants such as ascorbic acid, vitamin E, carotenoids, reduced glutathione, flavonoids and phenolic acids actually detected in fruits and vegetables of different varieties of cactus [55,22,56]. In addition, more recently, significant antioxidant properties of the most frequent cactus betalains have been revealed and numerous *in vitro *studies have demonstrated their ability to neutralize reactive oxygen species [21,57,25].

Oxidative stress is important as direct and indirect initiator as well as promoter of genotoxicity and apoptotic process. In order to elucidate the mechanism of genotoxic effect of AFB1, we have performed (i) the chromosome aberrations assay in bone marrow cells (ii) DNA fragmentation in liver and (iii) SOS Chromotest. Several studies have been conducted recently and have shown that AFB1 is a genotoxic agent. It has been shown that AFs especially AFB1 is activated by the hepatic cytochrome P450 enzyme system to produce a highly reactive intermediate, AFB1-8, 9-epoxide, which subsequently binds to nucleophilic sites in DNA and the major adduct 8, 9-dihydro-8-(N7guanyl)-9-hydroxy-AFB1 (AFB1 N7-Gua) is formed [11]. The formation of AFB1-DNA adducts is regarded as a critical step in the initiation of AFB1-induced hepatocarcinogenesis [58,59]. The above genotoxic endpoints are well known markers of genotoxicity and any reduction in the frequency of these genotoxic endpoints gives an indication of the antigenotoxicity of a particular compound [60]. In the current study, we tested the chromosomal aberrations assay which is widely used test to assess genotoxicity of chemicals. We have demonstrated that mice receiving AFB1 showed a high percentage of chromosome aberrations in their bone marrow cells (Figure 5); mainly breaks. It is acknowledged that an increase in this frequency is associated with an increased overall risk of cancer [61,62]. Most of the chromosomal aberrations observed in the cells are lethal, but there are many other aberrations that are viable and cause either somatic or inherited genetic effects [63]. There is a tendency for AFB1 to convert into the epoxide and produce DNA adducts that in turn cause DNA strand breaks and point mutations [64]. Mice pre and post-treated by CCE showed a significant reduction in the percentage of chromosome aberrations in their bone marrow cells and the protection was around 60% (Figure 5). To confirm the preventive effect of CCE against AFB1 genotoxicity, we looked for its eventual preventive effect against DNA fragmentation induced by AFB1. Indeed, we showed firstly that treatment with AFB1 (250 μg/kg b.w) induced a significant DNA fragmentation in liver cells of treated animals and no specific DNA fragments were observed for control. Simultaneous treatment of mice with AFB1 and CCE showed a significant restoration of DNA integrity (Figure 6). These results are in accordance with our recently published report involving preventive effect of CCE against genotoxicity induced by single intraperitonial treatment by the mycotoxin ZEN [28]. The protection, afforded by CCE against AFB1 genotoxicity is likely due to its ability to inhibit oxidative process induced by the mycotoxin AFB1. However, it could not be excluded that cactus extracts acts as antigenotoxic complex which enhances the DNA repair system or DNA synthesis. Among the studies performed in our laboratory we compared the prevention of ZEN genotoxic effects obtained by CCE to the prevention exerted by Vitamin E [65,66] and by a variety of hydrated sodium calcium aluminosilicate clay (HSCAS) [67] described as a compound able to adsorb and to sequester ZEN leading to the reduction of toxin bioavailability [68]. CCE appears clearly more efficient then Vitamin E and clay HSCAS.

The antigenotoxic activity of CCE was investigated in our study and the nongenotoxicity of this extract was checked. CCE may act, as described for other polyphenols such as flavonoids, by inhibiting microsomal activation or by directly protecting DNA strands from the electrophilic metabolite of mutagen compounds. They may inhibit several metabolic intermediates and reactive oxygen species (ROS) formed during the process of microsomal enzyme activation which are capable of breaking DNA strands [69,70].

The absence of genotoxicity is not a characteristic of all natural products in use; since other medicinal plants, tested with the SOS chromotest either in the presence or in the absence of the S9 preparation, have shown a genotoxic potential [71]. These tests showed that AFB1 present a genotoxic effect and that the treatment with CCE is able to diminish this genotoxicity (table 1).

After ingestion, AFB1 was shown to be converted into its epoxide and this derivative produces DNA adducts causing DNA strand breaks and point mutations [64]. Under this pathological condition, the active process of cellular self destruction, apoptosis may occur.

**Figure 10:**
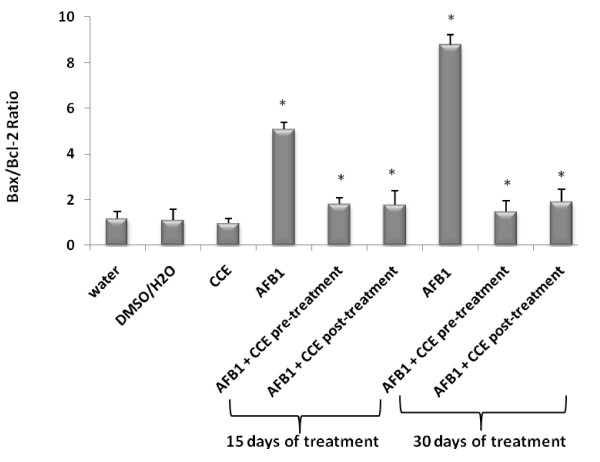
**Bax/Bcl-2 expression ratio in liver of Balb/c mice exposed to AFB1 (250 μg/Kg b.w.) for 15 days then 30 days and prevention by cactus cladode extract (50 mg/Kg b.w) before or after AFB1 administration**. Results were expressed as means ± S.D. from independent experiments. (*) indicated significant difference (p < 0.05) from control.

In the present study, the modulator effect of CCE on AFB1 toxicity was suggested to could be attributed to some alterations in the cell death pathway. P53 and Bax/Bcl-2 ratios play an important role in determining whether cells will undergo apoptosis (Figure 7, Figure 10).

Our results showed that treatment by AFB1 for 15 and 30 days induced higher expressions of p53 and bax pro-apoptotic proteins in liver tissues of AFB1 treated mice than in control groups. The same treatment induced a down-regulation of the antiapoptotic protein bcl2 (Figures 7a, b, 8a, b and 9a, b). Similarly to our results, Ranchal et al. (2009) [72] reported that AFB1 induced DNA damage, reduced p27 expression and increased cell death in cultured hepatocytes. Meanwhile, the CCE treatment before or after AFB1, has been shown to induce an anti-apoptotic effect via inhibition of p53 and bax expression (Figures 7a, b, 8a, b and 9a, b). This indicates that CCE can modulate the p53 dependent apoptotic pathway to restrict AFB1 toxicity.

The involvement of AFB1 in DNA damage and its correlation with biomarkers of cellular oxidative stress and apoptosis induction were also evaluated in our work. Oxidative stress can be considered as an apoptosis inducer [73]. Many agents that induce apoptosis are either oxidants or stimulators of cellular oxidative metabolism. This is the case of AFB1which induced oxidative stress and apoptotic cell death.

It is concluded that CCE is hepatoprotective as it enhances the activities of liver function, as evidenced by the decrease in MDA, protein carbonyls generation and Hsp 70 and Hsp 27 levels, it showed a total reduction of AFB1 induced genotoxicity markers and decrease the expressions of pro-apoptotic proteins p53 and bax. The mode of action of CCE might be preventing or scavenging the formation of ROS. Therefore, this plant should be considered as an accessible source of natural antioxidants.

Our results are perfectly in coherence with other published reports, which underlined the relevant preventive potential of cactus extracts [74]. It could be very interesting to include the cactus pear in food diet. However, more investigations are needed to unveil the protective potential of cactus cladodes and to identify the specific therapeutic agents present in this plant.

## List of abbreviations

CCE: Cactus cladode extract; AFB1: Aflatoxin B1; MDA: Malondialdehyde level; PC: Protein carbonyls; Hsp 70: Heat shock proteins 70; Hsp 27: Heat shock proteins 27; HCC: Hepatocellular carcinoma; AF: Aflatoxins; IF: Induction factor; β-gal: β- galactosidase; AP: Alkaline phosphatise; HSCA: Hydrated sodium calcium aluminosilicate; ROS: Reactive oxygen species; ZEN: Zearalenon.

## Competing interests

The authors declare that they have no competing interests.

## Authors' contributions

DB carried out the studies, acquired the data, performed the data analysis, and drafted the manuscript. CB and YA played a major role in the experimental procedures of this study and revised the manuscript. HbM carried out the part of genotoxicity tests; LZ carried out statistical analysis; HB involved in the design and organization of the study, interpreted the results and revised the manuscript. All authors have read and approved the final manuscript.

## References

[B1] Parkin DM Bray F Ferlay J Pisani P Global cancer statistics 2002 CA Cancer J Clin 2005 55 74 108 10.3322/canjclin.55.2.74 15761078

[B2] Jemal A Siegel R Ward E Hao Y Xu J Thun MJ Cancer statistics CA Cancer J Clin 2009 59 225 49 10.3322/caac.20006 19474385

[B3] Okuda K Hepatocellular carcinoma J Hepatol 2000 32 225 37 10728807 10.1016/s0168-8278(00)80428-6

[B4] Bartsch H Montesano R Relevance of nitrosamines to human cancer J Carcinog 1984 5 1381 93 10.1093/carcin/5.11.1381 6386215

[B5] Ribes J Cleries R Esteban L Moreno V Bosch FX The influence of alcohol consumption and hepatitis B and C infections on the risk of liver cancer in Europe J Hepatol 2008 49 233 42 10.1016/j.jhep.2008.04.016 18571275

[B6] Bennett JW Klich M Mycotoxins Clin Microbiol Rev 2003 16 497 516 10.1128/CMR.16.3.497-516.2003 12857779 PMC164220

[B7] Fink-Gremmels J Mycotoxins: their implications for human and animal health Vet Q& AArhive 1999 21 115 120 10568000 10.1080/01652176.1999.9695005

[B8] Cullen JM Newberne PM Eaton DL, Groopman JD Acute hepatotoxicity of aflatoxins The Toxicology of Aflatoxins: Human Health, Veterinary, and Agricultural Significance 1993 Academic Press, London

[B9] International Agency for Research on Cancer (IARC) Some traditional herbal medicines, some mycotoxins, naphthalene and styrene IARC Monographs on the Evaluation of Carcinogenic Risks to Humans 2002 82 1 556 12687954 PMC4781602

[B10] Roberts TA Baird-Parker AC Tompkin RB Toxigenic fungi: Aspergillus. Microorganisms in Food. 5 Microbiological Specification of Food Pathogens-ICMSF 1996 Chapter 19 Blackie Academic and Professional, London 347 38

[B11] Sharma RA Farmer PB Biological relevance of adduct detection to the chemoprevention of cancer Clin Cancer Res 2004 10 4901 4912 10.1158/1078-0432.CCR-04-0098 15297390

[B12] Klein PJ Van Vleet TR Hall JO Coulombe RA Jr Biochemical factors underlying the age-related sensitivity of turkeys to aflatoxin B (1) Comp Biochem Physiol part C 2002 132 193 201 10.1016/S1095-6433(01)00547-5 12106896

[B13] Shu Yuan Zhong-Wel Zhang Xu Fei Yang Hui Chen Yang-ER Yuan Ming Xu Mo-Yun Xue Li-Wei Xu Xiao-Chao Hong-Huilin Mg-protoporphyrin, haem and sugar signals double cellular total RNA against herbicide and high-light-derived oxidative stress Plant Cell Environ Plant 2011 in press 10.1111/j.1365-3040.2011.02302.x 21388419

[B14] Souza MF Tome AR Rao VS Inhibition by the bioflavonoid ternatin of aflatoxin B1-induced lipid peroxidation in rat liver J Pharm Pharmacol 1999 51 125 129 10217309 10.1211/0022357991772222

[B15] Okuno M Kojima S Moriwaki H Chemoprevention of hepatocellular carcinoma: concept, progress and perspectives J Gastroenterol Hepatol 2001 6 1329 35 10.1046/j.1440-1746.2001.02634.x 11851828

[B16] Kensler TW Quian GS Chen JG Groopman JD Translational strategies for cancer prevention in liver J Natl Cancer Inst 2003 3 321 9 10.1038/nrc1076 12724730

[B17] Heber D Vegetables, fruits and phytoestrogens in the prevention of diseases J Postgrad Med 2004 50 145 149 15235216

[B18] Acevedo A Fonseca E Viguer JM Contreras F Use of the peroxidise antiperoxidase technic in cytologic smears in pemphigus vulgaris Med Cutan Ibero Lat Am 1985 13 237 241 3906315

[B19] Stintzing FC Stintzing AS Carle R Frei B Wrolstad RE Color and antioxidant properties of cyanidin-based anthocyanin pigments J Agric Food Chem 2002 50 6172 6181 10.1021/jf0204811 12358498

[B20] Ramadan MF Morsel JT Oil cactus pear (*Opuntia ficus-indica*) Food Chem 2003 82 339 345 10.1016/S0308-8146(02)00550-2

[B21] Stintzing FC Carle R Cactus stems (Opuntia spp.): a review on their chemistry, technology, and uses Mol Nutr Food Res 2005 49 175 194 10.1002/mnfr.200400071 15729672

[B22] Tesoriere L Fazzari M Allegra M Livrea MA Biothiols, taurine, and lipid-soluble antioxidants in the edible pulp of Sicilian cactus pear (Opuntia ficus-indica) fruits and changes of bioactive juice components upon industrial processing J Agric Food Chem 2005 20 7851 7855 10.1021/jf050636f 16190641

[B23] Galati EM Mondello MR Giuffrida D Dugo G Miceli N Pergolizzi S Taviano MF Chemical characterization and biological effects of Sicilian Opuntiaficusindica (L.) Mill. fruit juice: antioxidant and antiulcerogenic activity Agric Food Chem 2003 51 4903 4908 10.1021/jf030123d 12903943

[B24] Kuti JO Antioxidant compounds from four Opuntia cactus pear fruit varieties Food Chem 2004 85 527 533 10.1016/S0308-8146(03)00184-5

[B25] Tesoriere L Butera D Pintaudi M Allegra M Livrea MA Supplementation with cactus pear (Opuntiaficus-indica) fruit decreases oxidative stress in healthy humans: a comparative study with Vit C Am J Clin Nutr 2004 80 391 395 10.1093/ajcn/80.2.391 15277160

[B26] Zou DM Brewer M Garcia F Feugang JM Wang J Zang R Liu H Zou C Cactus pear: anatural product in cancer chemoprevention Nutrition Journal 2005 4 25 36 10.1186/1475-2891-4-25 16150152 PMC1242252

[B27] Galati EM Mondello MR Lauriano ER Traviano MF Galluzzo M Miceli N Opuntiaficus-indica (L.) Miller fruit juice protects liver from carbon tetrachloride induced injury Phytother Res 2005 19 796 800 10.1002/ptr.1741 16220574

[B28] Zourgui L Imen AB Yosra A Hassen B Wafa H The antigenotoxic activities of cactus (Opuntiaficus-indica) cladodes against the mycotoxinzearalenone in Balb/c mice: prevention of micronuclei, chromosome aberrations and DNA fragmentation Food Chem Toxicol 2009 47 662 667 10.1016/j.fct.2008.12.031 19152824

[B29] Bradford MM A rapid and sensitive method for quantitation of microgram quantities of protein utilizing the principle of protein-dye-binding Anal Biochem 1976 72 248 54 10.1016/0003-2697(76)90527-3 942051

[B30] Aust SD Morehouse LA Thomas CE Role of metals in oxygen radical reactions Journal of Free Radicals in Biology and Medicine 1985 1 3 25 10.1016/0748-5514(85)90025-X 3013969

[B31] Mercier Y Gatellier P Renerre M Lipid and protein oxidation in vitro, and antioxidant potential in meat from Charolais cows finished on pasture or mixed diet Meat Sci 2004 66 467 473 10.1016/S0309-1740(03)00135-9 22064150

[B32] Evans EP Breckon G Ford CE An air drying method for meiotic preparation from mammalian tests Cytogenet 1960 3 613 616 10.1159/000129818 14248459

[B33] Savage JRK Classification and relationships of induced chromosomal structural changes J Med Genet 1975 12 103 122 10.1136/jmg.13.2.103 PMC1013369 933108

[B34] Maron DM Ames BN Revised methods for the *Salmonella *mutagenicity test Mutat Res 1983 113 173 215 6341825 10.1016/0165-1161(83)90010-9

[B35] Quillardet P Hofnung M The SOS Chromotest, a colorimetric bacterial assay for genotoxins: procedures Mutat Res 1985 147 65 78 3923333 10.1016/0165-1161(85)90020-2

[B36] Roebuck BD Liu YL Rogers AE Groopman JD Kensler TW Protection against aflatoxin B1-induced hepatocarcinogenesis in F344 rats by 5-(2-pyrazinyl)-4-methyl-1,2-dithiole-3-thione (oltipraz): predictive role for short-term molecular dosimetry Cancer Res 1991 51 5501 5506 1680553

[B37] Cullen JM Newberne PM Eaton DL, Groopman JD Acute hepatotoxicity of aflatoxins Toxicol Aflatoxins 1994 San Diego, California: Academic Press 3 26

[B38] Roebuck BD Maxuitenko Y Eaton DL, Groopman JD Biochemical mechanisms and biological implications of the toxicity of aflatoxins as related to aflatoxin carcinogenesis The Toxicology of Aflatoxins: Human Health, Veterinary, and Agricultural Significance 1994 New York: Academic Press 27 43

[B39] Kim HS Kwack SJ Lee BM Lipid peroxidation, antioxidant enzymes, and benzo[a]pyrene-quinones in the blood of rats treated with benzo[a]pyrene Chem Biol Interact 2000 127 139 150 10.1016/S0009-2797(00)00177-0 10936229

[B40] Dotan Y Lichtenberg D Pinchuk I Lipid peroxidation cannot be used as a universal criterion of oxidative stress Prog Lipid Res 2004 43 200 227 10.1016/j.plipres.2003.10.001 15003395

[B41] Preetha SP Kanniappan M Selvakumar E Nagaraj M Varalakshmi P Lupeol ameliorates aflatoxin B1-induced peroxidative hepatic damage in rats Comp Biochem Physiol C Comp Pharmacol 2006 143 333 339 10.1016/j.cbpc.2006.03.008 16730236

[B42] Amstad P Levy A Emerıt I Ceruttı P Evidence for membrane mediated chromosomal damage by aflatoxin b1 in human lymphocytes Carcinogenesis 1984 5 719 723 10.1093/carcin/5.6.719 6426812

[B43] Abdel-Wahhab MA Aly SE Antioxidants and radical scavenging properties of vegetable extracts in rats fed aflatoxin-contaminated diet J Agric Food Chem 2003 51 2409 2414 10.1021/jf0209185 12670189

[B44] Dalle-Donne I Giustarini D Colombo R Rossi R Milzani A Protein carbonylation in human diseases Trends Mol Med 2003 9 169 176 10.1016/S1471-4914(03)00031-5 12727143

[B45] Dalle-Donne I Rossi R Giustarini D Milzani A Colombo R Protein carbonyl groups as biomarkers of oxidative damage in human disease Clin Chim Acta 2003 329 23 38 10.1016/S0009-8981(03)00003-2 12589963

[B46] Okada S Iron-induced tissue damage and cancer: the role of reactive oxygen species-free radicals Pathol Int 1996 46 311 332 10.1111/j.1440-1827.1996.tb03617.x 8809878

[B47] Ritossa F A new puffing pattern induced by temperature shock and DNP in Drosophila Experienta 1962 18 571 573 10.1007/BF02172188

[B48] Welch WJ How cells respond to stress Sci Am 1993 5 560 564 10.1038/scientificamerican0593-56 8097593

[B49] Voellmy R Feige U, Morimoto RI, Yahara I, Polla B Sensing stress and responding to stress Stress Inducible Cellular Responses 1996 Birkhäuser-Verlag, Basel, Switzerland 121 137

[B50] Zou J Salminen WF Roberts SM Voellmy R Correlation between glutathione oxidation and trimerization of heat shock factor 1, an early step in stress induction of the Hsp response Cell Stress Chaprones 1998 3 130 141 10.1379/1466-1268(1998)003<0130:CBGOAT>2.3.CO;2 PMC312956 9672248

[B51] Freeman ML Borelli MJ Meredith MJ Lepock JR On the path to the heat shock response: destabilisation and formation of partially folded protein intermediates, a consequence of protein thiol modification Free Radic Biol Med 1999 26 737 745 10.1016/S0891-5849(98)00258-5 10218664

[B52] Beyersmann D Hechtenberg S Cadmium, gene regulation, and cellular signaling in mammalian cells Toxicol Appl Pharmacol 1997 144 247 261 10.1006/taap.1997.8125 9194408

[B53] Fehrenbach E Northoff H Free radicals exercise apoptosis and heat shock proteins Immunol Rev 2001 7 66 89 11579749

[B54] Zourgui L Golli EE Bouaziz C Bacha H Hassen W Cactus (Opuntia ficus indica) Cladodes prevent oxidative damage induced by the mycotoxin zearalenone in Balb/c mice Food Chem Toxicol 2008 46 1817 1824 10.1016/j.fct.2008.01.023 18313193

[B55] Park EH Kahng JH Paek EA Studies on the pharmacological actions of cactus: identification of is anti-inflammatory effect Arch Pharm Res 1998 21 30 34 10.1007/BF03216749 9875511

[B56] Shim HC Hwang HJ Kang KJ Lee BH An antioxidative and anti inflammatory agent for potential treatment of osteoarthritis from Ecklonia cava Arch Pharm Res 2006 29 165 171 10.1007/BF02974279 16526282

[B57] Siriwardhana N Jeon YJ Antioxydative effect of cactus pear fruit (Opuntiaficusindica) extract on lipid peroxidation inhibition in oils and emulsion model systems Eur Food Res Technol 2004 219 369 376

[B58] Preston RJ Williams GM DNA-reactive carcinogens: mode of action and human cancer hazard Crit Rev Toxicol 2005 35 673 683 10.1080/10408440591007278 16417034

[B59] Abdel-Wahhab MA Ahmed HH Hagazi MM Prevention of aflatoxin B1-initiated hepatotoxicity in rat by marine algae extracts J Appl Toxicol 2006 26 229 38 10.1002/jat.1127 16389658

[B60] Albertini RJ Ardell SK Judice SA Jacobson S Allegretta M Hypoxanthine-guanine phosphoribosyltransferase reporter gene mutation for analysis of in vivo clonal amplification in patients with HTLV type 1-associated Myelopathy/Tropical spastic paraparesis AIDS Res Hum Retroviruses 2000 16 1747 1752 10.1089/08892220050193254 11080821

[B61] Hagmar L Brogger A Hansteen IL Heim S Högstedt B Knudsen L Lambert B Linnainmaa K Mitelman F Nordenson I Cancer risk in humans predicted by increased levels of chromosomal aberrations in lymphocytes: nordic study group on the health risk of chromosome damage Cancer Res 1994 54 2919 2922 8187078

[B62] Hagmar L Bonassi S Strömberg U Mikoczy Z Lando C Hansteen IL Montagud AH Knudsen L Norppa H Reuterwall C Tinnerberg H Brogger A Forni A Högstedt B Lambert B Mitelman F Nordenson I Salomaa S Skerfving S Cancer predictive value of cytogenetic markers used in occupational health surveillance programs: a report from an ongoing study by the European Study Group on Cytogenetic Biomarkers and Health Mutat Res 1998 405 171 178 10.1016/S0027-5107(98)00134-1 9748557

[B63] Swierenga SH Heddle JA Sigal EA Gilman JP Brillinger RL Douglas GR Nestmann ER Recommended protocols based on a survey of current practice in genotoxicity testing laboratories, IV. Chromosome aberration and sister-chromatid exchange in Chinese hamster ovary, V79 Chinese hamster lung and human lymphocyte cultures Mutat Res 1991 246 227 233 10.1016/0027-5107(91)90046-Q 1996126

[B64] Eaton DL Gallagher EP Mechanisms of aflatoxin carcinogenesis Annu Rev PharmacolToxicol 1994 34 135 172 10.1146/annurev.pa.34.040194.001031 8042848

[B65] Abid-Essefi S Baudrimont I Hassen W Ouanes Z Mobio TA Anane R Creppy E Bacha H DNA fragmentation, apoptosis and cell cycle arrest induced by zearalenone in cultured DOK, Vero and Caco-2 cells: prevention by vitamin E Toxicol 2003 192 237 248 10.1016/S0300-483X(03)00329-9 14580790

[B66] Ouanes Z Ayed-Boussema I Baati T Creppy EE Bacha H Zearalenone induces chromosome aberrations in mouse bone marrow: preventive effect of 17b estradiol, progesterone and Vitamine E Mutat Res 2005 565 139 149 15661612 10.1016/j.mrgentox.2004.10.005

[B67] Abbès S Ouanes Z Ben Salah-Abbes J Houas Z Oueslati R Bacha H Othman O The protective effect of hydrated sodium calcium aluminosilicate against haematological biochemical and pathological changes induced by zearalenone in mice Toxicon 2006 47 567 574 10.1016/j.toxicon.2006.01.016 16563452

[B68] Phillips TD Dietary clay in the chemoprevention of aflatoxin induced disease Toxicol Sci 1999 52 118 126 10630600 10.1093/toxsci/52.suppl_1.118

[B69] De Flora S Problems and prospects in antimutagenesis and anticarcinogenesis Mutat Res 1988 202 279 83 10.1016/0027-5107(88)90192-3 3057361

[B70] Shon MY Choi SD Kahng GG Nam SH Sung NJ Antimutagenic, antioxidant and free radical scavenging activity of ethyl acetate extracts from white, yellow and red onions Food Chem Toxicol 2004 42 659 666 10.1016/j.fct.2003.12.002 15019191

[B71] De Carvalho MCRD Barca FNTV Agnez-Lima LF de Medeiros SRB Evaluation of mutagenic activity in an extract of pepper tree stem bark (*Schinus terebinthifolius *Raddi) Enviro Mol Mutagen 2003 42 185 191 10.1002/em.10183 14556225

[B72] Ranchal I Gonzalez R Bello RI Ferrin G Hidalgo AB Linares CI Aguilar Melero P Gonzalez Rubio S Barrera P Marchal T Nakayama KI de la malta M Muntane J The reduction of cell death and proliferation by P27 (Kip1) minimizes DNA damage in an experimental model of genotoxicity Int J Cancer 2009 125 2270 2280 10.1002/ijc.24621 19672859

[B73] Chandra J Samali A Orrenius S Triggering and modulation of apoptosis by oxidative stress Free Radical Biol Med 2000 29 323 333 10.1016/S0891-5849(00)00302-6 11035261

[B74] Feugang JM Konarski P Zou D Stintzing FC Zou C Nutritional and medicinal use of cactus pear (Opuntia spp.) cladodes and fruits Frontiers Biosci 2006 11 2574 2586 10.2741/1992 16720335

